# The airway epithelial–immune axis: mechanisms and therapeutic implications

**DOI:** 10.3389/fimmu.2026.1891760

**Published:** 2026-07-24

**Authors:** Zhengyi Zhu, Qiang Li, Binjian Hua

**Affiliations:** 1 Department of Hematology, Central Hospital of Haining, Jiaxing, Zhejiang, China; 2 Department of Respiratory Medicine, Jiaxing Hospital of Traditional Chinese Medicine, Jiaxing, Zhejiang, China

**Keywords:** airway epithelium, alarmins, barrier dysfunction, chronic obstructive pulmonary disease, epithelial-immune axis, precision medicine

## Abstract

The airway epithelium is increasingly recognized not merely as a physical barrier, but as a central, active regulator of mucosal immunity. This review comprehensively summarizes the structural and functional basis of the airway epithelial-immune axis and its critical role in chronic respiratory diseases. Exposure to environmental allergens, pollutants, and respiratory pathogens disrupts epithelial tight junctions and triggers the rapid release of key alarmins, including TSLP, IL-33, and IL-25. These epithelial-derived cytokines participate in reciprocal epithelial-immune circuits, driving extensive crosstalk with both innate (ILC2s) and adaptive (Th2 cells) immune networks to establish self-perpetuating inflammatory loops. Such epithelial dysfunction can act as an important driver and amplifier in the pathogenesis of asthma, chronic obstructive pulmonary disease (COPD), and upper airway inflammatory disorders. Consequently, targeting this axis has emerged as a promising therapeutic strategy, shifting the focus toward alarmin-neutralizing biologics, upstream receptor inhibition, and barrier restoration. Furthermore, we highlight how emerging technologies—such as single-cell RNA sequencing, spatial transcriptomics, organoid models, and multi-omics integration—are decoding cellular heterogeneity and spatial niches, ultimately paving the way for precision medicine and long-term disease-modifying therapies in respiratory medicine.

## 1 Introduction

Chronic respiratory diseases, including bronchial asthma, chronic obstructive pulmonary disease (COPD), allergic rhinitis, and chronic rhinosinusitis, represent a substantial and growing global health burden (1, 2). According to estimates from the World Health Organization, asthma alone affects more than 300 million individuals worldwide, whereas COPD remains a leading cause of morbidity and mortality across diverse populations (3). The considerable socioeconomic burden, the high frequency of acute exacerbations, and the progressive and often irreversible decline in lung function collectively highlight the urgent need for a more comprehensive understanding of disease mechanisms and the development of more effective therapeutic strategies.

For decades, airway disease pathophysiology was viewed mainly through the lens of adaptive immunity. However, contemporary perspectives emphasize that epithelial barrier dysfunction and innate immune activation interact reciprocally with adaptive immune responses in chronic airway inflammation (4, 5). In earlier models, the airway epithelium was primarily regarded as a passive structural component, functioning mainly as a physical barrier responsible for regulating water and ion transport, while facilitating mucociliary clearance of inhaled particles (6). In this bidirectional framework, epithelial cells can initiate and amplify inflammation, while Th2 cells, B cells, IgE-mediated mast-cell activation, and immune memory can in turn maintain or worsen epithelial dysfunction.

In recent years, however, this traditional perspective has been fundamentally revised. Increasing evidence has demonstrated that the airway epithelium is not merely a structural barrier, but rather a highly dynamic and functionally versatile tissue involved in inflammation, immune regulation, host defense, and tissue remodeling (7–10). It is now widely accepted that the airway epithelium serves as an active regulator of innate immunity and acts as a critical interface between the external environment and the internal host milieu. Epithelial-derived mediators have been increasingly recognized as central contributors to the initiation and propagation of airway inflammation, particularly in asthma pathogenesis (3). As a result, the concept of an airway epithelial–immune axis has been established as a key framework for understanding the regulation of mucosal homeostasis and the development of airway diseases.

This epithelial–immune axis can be understood through three interrelated functional components, including the maintenance of epithelial barrier integrity, the capacity for immune sensing, and the role of the epithelium as an amplifier of inflammatory responses (11). The primary function of the airway mucosa is to maintain internal homeostasis by separating the host from environmental exposures (12–14). When this barrier is disrupted by environmental toxins, allergens, or pathogens, epithelial cells rapidly activate pattern recognition receptors (PRRs), leading to the release of a wide range of mediators, including alarmins, antimicrobial peptides, and chemokines. These mediators collectively regulate the intensity, duration, and phenotype of subsequent innate and adaptive immune responses. In this context, epithelial dysfunction has been increasingly linked to airway remodeling processes and the heterogeneity of disease phenotypes observed across different stages of life (15–17).

This review uses the airway epithelial-immune axis as a conceptual framework rather than a purely descriptive catalogue. We organize the literature around three linked questions: how epithelial structural defects arise, how injured epithelial cells translate environmental signals into immune programs, and which upstream nodes are most actionable therapeutically. Across these sections, we distinguish well-established mechanisms, such as tight-junction disruption and alarmin release, from unresolved issues, including disease-specific epithelial cell states, the relative value of barrier repair versus immune blockade, and the biomarkers needed to select patients for epithelial-targeted therapies.

## 2 Structural and functional basis of airway epithelium

### 2.1 Airway epithelial cell types and composition

**Figure 1:**
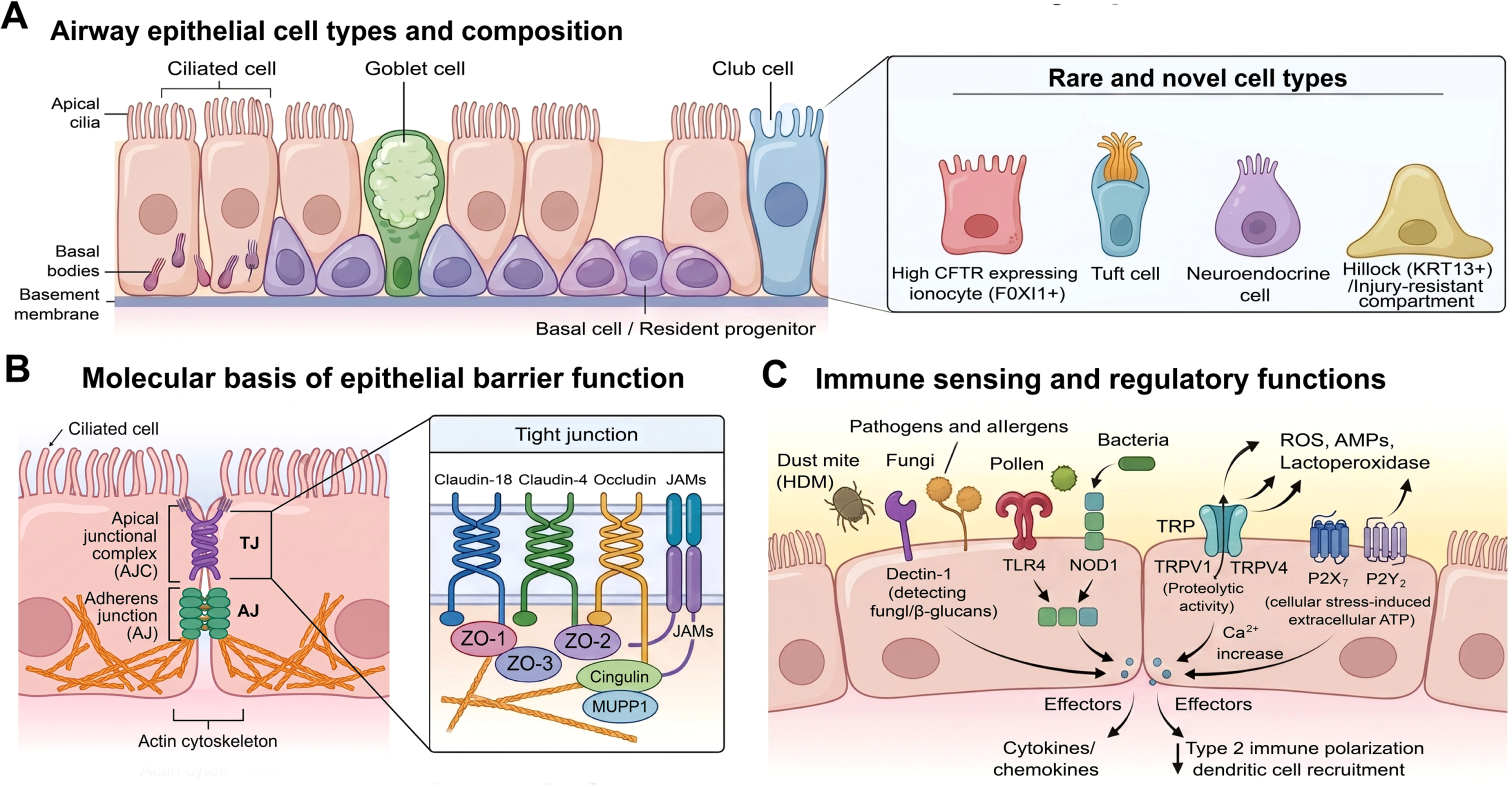
Structural and functional overview of the airway epithelium. **(A)** Cellular composition: the pseudostratified airway epithelium comprises classical populations (ciliated, goblet, club, and basal cells) and rare/novel cell types (ionocytes, tuft cells, neuroendocrine cells, and hillocks) that maintain tissue homeostasis. **(B)** Epithelial barrier: barrier integrity is maintained by apicolaterally positioned tight junctions and adherens junctions, which together form the AJC, while desmosomes provide additional lateral mechanical adhesion. The revised schematic depicts the actin cytoskeleton as an intracellular structure linked to junctional scaffolding proteins. Tight junctions consist of transmembrane proteins (claudins, occludin, JAMs) anchored to intracellular scaffolds (ZO-1/2/3, cingulin, MUPP1). **(C)** Immune sensing: Epithelial cells continuously monitor the environment. They detect allergens and pathogens via pattern recognition receptors (e.g., Dectin-1, TLR4, NOD1) and utilize sensory channels (e.g., TRPV1/4, P2X7/P2Y2) to integrate external stimuli. This triggers calcium influx and the secretion of antimicrobial effectors, cytokines, and chemokines to regulate downstream immune responses.

The airway epithelium exhibits a highly organized pseudostratified structure composed of multiple distinct yet functionally interconnected cell populations (**Table 1**). In the conducting airways, the classical cellular composition includes basal cells, club cells, ciliated cells, and mucus-secreting goblet cells (39, 40) (**Figure 1A**). Basal cells function as resident progenitor cells and are anchored to the basement membrane. They possess the capacity for self-renewal and multipotent differentiation, enabling them to regenerate the entire epithelial layer following injury or infection (39). Club cells, formerly termed Clara cells, are enriched within the distal conducting airways and contribute to epithelial homeostasis through xenobiotic detoxification, secretion of immunoregulatory proteins, and facultative progenitor activity during epithelial repair (41, 42). Ciliated cells occupy much of the apical epithelial surface and mediate mucociliary clearance via synchronized ciliary beating, thereby enabling efficient removal of mucus, pathogens, and inhaled particulates (43). Goblet cells specialize in the production of gel-forming mucins that establish a protective mucus barrier capable of trapping environmental agents and microorganisms (44).

**Table 1:** Role of epithelium-driven inflammation in respiratory diseases.

Disease category	Key epithelial alterations	Dominant immune mechanisms	Clinical & pathological features	References
Asthma	Impaired barrier integrity; disrupted tight junctions; increased baseline TSLP, IL-33, and IL-25.	Primarily Type 2 (eosinophilic) via alarmins; severe subset driven by T1/IFN-γ (neutrophilic).	Airway hyperresponsiveness (AHR); mucus overproduction; subepithelial fibrosis.	(18–22)
COPD	Senescence; altered differentiation (goblet cell hyperplasia, reduced ciliated cells); persistent TGF-β production.	Shift to Type 1-dominant; macrophage and neutrophil activation via altered IL-33/ST2 signaling.	Small airway fibrosis; progressive lung function decline (reduced FEV1); tissue damage.	(23–27)
Upper Airway Diseases *(Rhinitis, CRS)*	Barrier disruption; increased alarmin (TSLP) production linking upper and lower airways.	Type 2 amplification; local eosinophilic inflammation; systemic immunomodulation.	Tissue remodeling; nasal polyp formation; progression of inflammation to lower airways.	(28–32)
Viral Infections *(RSV, hMPV)*	Structural disruption; long-lasting epigenetic alterations in basal progenitor cells.	Release of IL-33, TSLP, and HMGB1; lower threshold for future Type 2 inflammation.	Early-life sensitization; recurrent wheezing; increased susceptibility to chronic respiratory diseases.	(33–38)

Recent advances in single-cell transcriptomic technologies have revealed a previously underappreciated level of cellular heterogeneity within the airway epithelium. Rare epithelial cell populations, including solitary neuroendocrine cells and tuft cells, have been identified and are increasingly recognized for their specialized roles in environmental sensing and the regulation of neuroimmune interactions, although their lineage relationships and precise functional contributions remain to be fully elucidated (45–47). In addition to these populations, novel epithelial cell types have been identified through high-resolution transcriptomic analyses. Among these, pulmonary ionocytes represent a rare cell population characterized by expression of FOXI1. These cells exhibit high levels of CFTR expression, suggesting a central role in regulating airway ion transport and fluid homeostasis (48). Furthermore, distinct epithelial structures termed hillocks, marked by expression of KRT13, SCEL, and SPRR1A/B, have been described. These structures appear to function as a specialized epithelial compartment with enhanced resistance to injury and the capacity to repopulate the airway epithelium following severe damage (49, 50) (**Figure 1A**).

### 2.2 Epithelial barrier function and its molecular basis

The airway epithelial barrier is maintained by a multilayered defense system that tightly regulates the passage of solutes, pathogens, and environmental agents. The principal components of this system include mucociliary clearance, intercellular junctional complexes, and the secretion of antimicrobial factors (51).At the cellular level, barrier integrity is supported by the apical junctional complex (AJC), which is composed principally of tight junctions (TJs) and adherens junctions (AJs), together with additional adhesive structures that provide mechanical resilience. TJs and AJs occupy the apicolateral domain, regulate paracellular permeability, and connect junctional proteins to the actin cytoskeleton through adaptor proteins (52) (**Figure 1B**). Desmosomes are distinct from the AJC because they are positioned more laterally and link cadherin-family proteins to intermediate filaments rather than to actin. Nevertheless, they complement TJs and AJs by strengthening intercellular adhesion and altered desmosomal junctions have been implicated in inflammatory airway and sinonasal epithelial disease (53).

Tight junctions are localized at the apicolateral membrane and play a central role in regulating paracellular permeability. Their molecular architecture consists of transmembrane proteins, including members of the claudin family, occludin, and junctional adhesion molecules, which interact across the intercellular space. Intracellularly, these proteins are anchored to cytoskeletal elements via scaffolding proteins such as zonula occludens (ZO-1, ZO-2, ZO-3), cingulin, and MUPP1 (54, 55) (**Figure 1B**). Specific claudin isoforms contribute differentially to barrier function. Claudin-18 is closely associated with epithelial barrier integrity and has been shown to be reduced in individuals with asthma, indicating a potential role in disease susceptibility (21). Claudin-4 is involved in the regulation of paracellular sodium permeability and has been correlated with the severity of airway inflammation (56). Disruption of these junctional complexes compromises epithelial integrity, leading to increased permeability. This facilitates the translocation of environmental agents into subepithelial compartments, thereby promoting the activation of local immune responses.

Clinical studies provide direct support for defective airway epithelial barrier function in asthma. Bronchial epithelial cells obtained from patients with asthma show impaired barrier formation, increased epithelial fragility, and altered repair responses compared with cells from healthy controls (57). These patient-derived observations complement mechanistic studies linking reduced junctional proteins, including claudin-18, to increased epithelial permeability and airway hyperresponsiveness (21). Recent experimental evidence also indicates that NLRP3 contributes to epithelial barrier homeostasis: NLRP3 deficiency reduced the expression of barrier-associated molecules, including Cldn18, Tjp1, and E-cadherin, increased allergen uptake by lung conventional dendritic cells, and enhanced airway hyperresponsiveness in allergic airway inflammation (22).

### 2.3 Immune sensing and regulatory functions

The airway epithelium functions as an active interface that continuously monitors the luminal environment through a highly specialized array of sensory mechanisms. Upon exposure to environmental stimuli, epithelial cells detect pathogen-associated molecular patterns (PAMPs) and damage-associated molecular patterns (DAMPs), leading to the production of cytokines and chemokines that promote type 2 immune polarization and recruit dendritic cells (58). This surveillance process is primarily mediated by pattern recognition receptors (PRRs), including Toll-like receptors (TLRs), NOD-like receptors (NLRs), and C-type lectin receptors (CLRs) (3). For instance, the C-type lectin receptor Dectin-1 specifically recognizes β-glucan structures in the cell walls of inhaled fungi such as *Aspergillus fumigatus*. It also contributes to immune responses against complex environmental allergens like animal dander, pollen, cockroach allergens, and house dust mite (HDM) extracts, potentially by sensing contaminating fungal components or via indirect signaling pathways (59, 60). In parallel, activation of TLR4 by HDM-associated microbial components (e.g., LPS)​ provides a rapid trigger for downstream inflammatory signaling pathways, while CLRs (such as Dectin-2) directly sense HDM-derived components. Additionally, intracellular NLRs such as NOD1/NOD2 detect bacterial peptidoglycan fragments, which may originate from microbial contaminants in the environment and contribute to Th2 polarization (61, 62) (**Figure 1C**). The NLR family also includes NLRP3, which links epithelial immune sensing with barrier regulation; recent evidence suggests that NLRP3-dependent pathways may support airway epithelial barrier homeostasis rather than functioning solely as pro-inflammatory inflammasome signals (22).

In addition to classical PRR-mediated sensing, airway epithelial cells utilize transient receptor potential (TRP) channels, particularly calcium-permeable channels such as TRPV1 and TRPV4, to detect proteolytic activity derived from common aeroallergens, including *Alternaria alternata* and HDMs (63, 64). Allergen exposure also induces cellular stress responses that result in the release of extracellular ATP, which functions as a damage-associated molecular signal. Extracellular ATP subsequently activates purinergic receptors, including P2X_7_ and P2Y_2_, leading to increased intracellular calcium levels and amplification of downstream signaling pathways (65) (**Figure 1C**). Activation of these sensory systems initiates a series of intracellular signaling cascades that promote the transcription and secretion of effector molecules, including reactive oxygen species (ROS) and antimicrobial peptides (AMPs). In addition, epithelial cells produce antimicrobial enzymes such as lactoperoxidase, which contributes to the generation of oxidized antimicrobial products (66). Through these mechanisms, the airway epithelium integrates diverse environmental signals and converts them into coordinated biochemical responses that regulate both innate and adaptive immunity.

## 3 Mechanisms of airway epithelial injury and barrier disruption

### 3.1 Environmental and pathogen-mediated epithelial injury

The respiratory mucosa is continuously exposed to a large volume of inhaled air, estimated at approximately 10,000 to 20,000 liters per day (67). This extensive exposure places the epithelial layer in direct contact with a wide range of environmental challenges, including allergens, respiratory viruses, bacteria, and particulate pollutants. Environmental allergens, such as those derived from house dust mites, fungi, and pollens, often exhibit intrinsic proteolytic activity that directly compromises epithelial integrity (32). Proteases contained within these allergens disrupt both tight junctions and adherens junctions and can activate signaling pathways including epidermal growth factor receptor (EGFR) and Wnt/β-catenin pathways, thereby contributing to structural damage of the epithelial barrier (51, 68, 69). In addition, exposure to particulate matter, including fine particles such as PM2.5, as well as cigarette smoke, induces pronounced oxidative stress. This process leads to epithelial cytotoxicity, lipid peroxidation, and loss of epithelial coverage, ultimately impairing mucociliary clearance (70).

**Figure 2:**
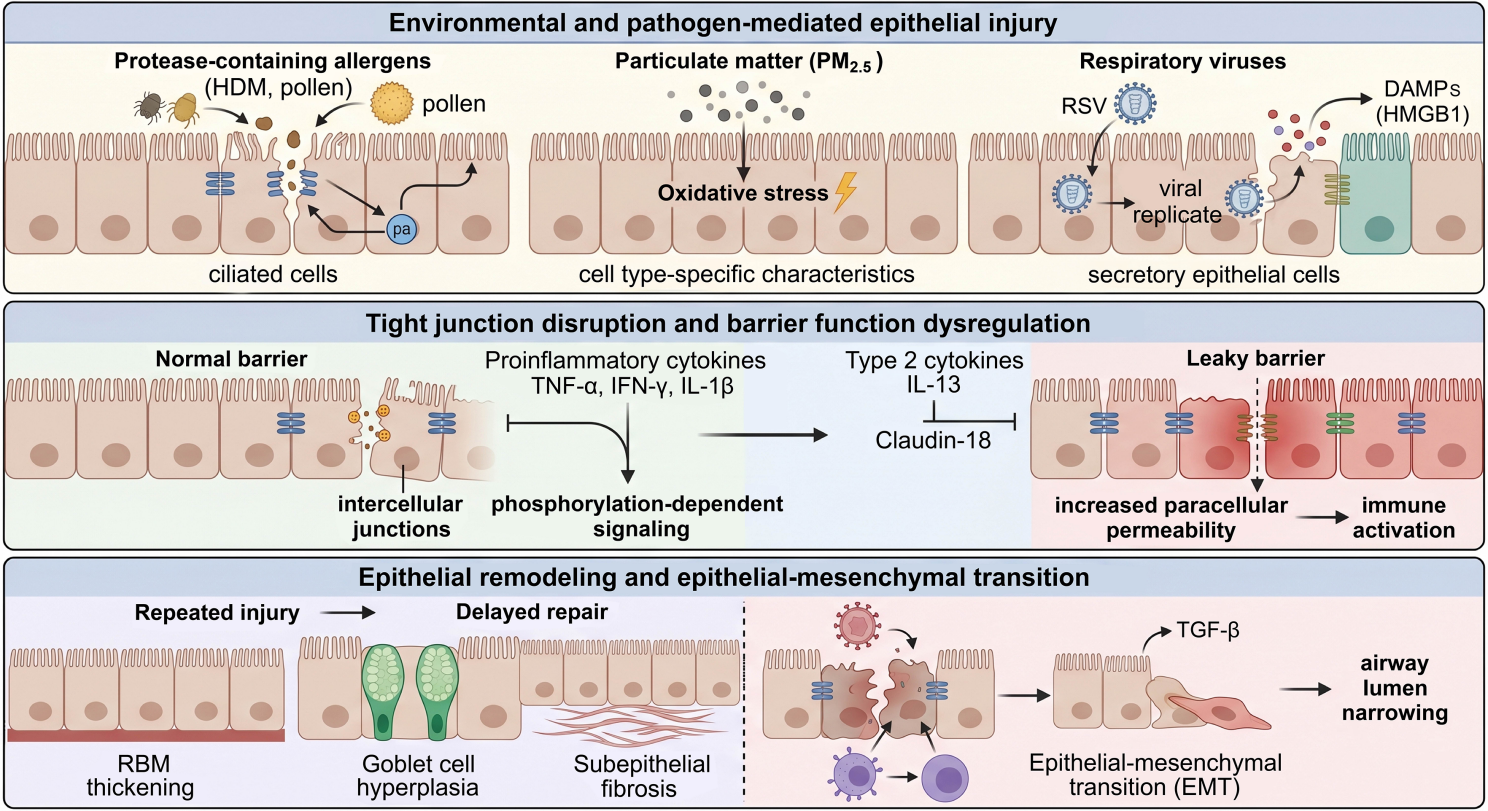
Mechanisms of airway epithelial injury, barrier disruption, and remodeling. (Top) epithelial injury: protease-containing allergens, PM2.5-induced oxidative stress, and respiratory viruses (e.g., RSV) damage the epithelium and trigger the release of DAMPs (e.g., HMGB1). (Middle) barrier disruption: proinflammatory and type 2 cytokines (e.g., IL-13) impair junctional signaling and downregulate key proteins like Claudin-18, creating a “leaky barrier” with increased permeability. (Bottom) structural remodeling: repeated injury and delayed repair lead to RBM thickening, goblet cell hyperplasia, subepithelial fibrosis, and TGF-β-driven epithelial-mesenchymal transition (EMT), culminating in airway lumen narrowing.

Respiratory pathogens also play a critical role in epithelial injury, particularly in early life, where infections may influence long-term disease susceptibility. Viruses such as respiratory syncytial virus (RSV), human rhinovirus (HRV), and human metapneumovirus (hMPV) utilize epithelial cells as primary targets for replication (33, 71, 72). RSV infection induces substantial structural damage and promotes the release of damage-associated molecular patterns (DAMPs), including high mobility group box 1 (HMGB1). Experimental evidence indicates that RSV-induced reactive oxygen species (ROS) production enhances the release of HMGB1 from infected epithelial cells, thereby amplifying downstream inflammatory responses and promoting monocyte activation (34, 35, 73) (**Figure 2**).

Host epithelial responses to pathogens may also exhibit cell type–specific characteristics. For example, single-cell transcriptomic analyses have shown that Aspergillus fumigatus preferentially affects ciliated cells, inducing stress responses related to protein folding, whereas Coccidioides posadasii infection triggers hypoxia-associated transcriptional programs in secretory epithelial cells (74). Collectively, these environmental and pathogen-derived insults exceed the detoxification capacity of club cells and impair the mechanical clearance function of ciliated cells. This imbalance contributes to sustained epithelial injury and promotes the development of chronic barrier dysfunction.

### 3.2 Tight junction disruption and barrier function dysregulation

A persistent increase in epithelial permeability is a common feature of chronic respiratory diseases and reflects disruption of barrier integrity. Although epithelial permeability is widely recognized as a characteristic of mucosal inflammation, the underlying molecular mechanisms are still being elucidated (52, 75, 76). Current evidence indicates that disruption of intercellular junctions occurs both as a direct consequence of epithelial injury and as a process that further amplifies immune activation (77). Proinflammatory cytokines within the airway microenvironment can directly disrupt epithelial junctional integrity. For example, IL-1β has been shown to modulate transepithelial ion permeability and alter tight junction function in epithelial monolayers (78). In addition, combined exposure to tumor necrosis factor-α (TNF-α) and interferon-γ (IFN-γ) induces significant impairment of tight junction barrier function (78). These effects can be attenuated by inhibition of protein kinase C, indicating that phosphorylation-dependent signaling pathways play a central role in regulating junctional disassembly (78, 79).

In type 2 (T2)-high asthma, the cytokine milieu further disrupts epithelial barrier integrity by suppressing the expression of key junctional proteins. Transcriptomic analyses have demonstrated that IL-13–mediated downregulation of claudin-18 is associated with increased epithelial permeability, enhanced sensitization to aeroallergens, and airway hyperresponsiveness (21, 80, 81) (**Figure 2**). Loss of junctional integrity leads to increased paracellular permeability, allowing allergens, microbial components, and pollutants to penetrate the epithelial barrier and access subepithelial immune cells, including dendritic cells, macrophages, and mast cells (82). This continuous exposure sustains immune activation and contributes to chronic inflammation. Accumulating evidence therefore supports the concept that epithelial barrier dysfunction represents a central mechanism underlying type 2 immune responses in airway disease (31). Importantly, inflammasome-associated pathways can also exert context-dependent homeostatic functions. In experimental allergic airway inflammation, NLRP3 deficiency was associated with reduced Cldn18, Tjp1, and E-cadherin expression, increased epithelial leakiness and allergen capture by lung conventional dendritic cells, and enhanced airway hyperresponsiveness, indicating that NLRP3 may help preserve epithelial barrier integrity under selected conditions (22).

### 3.3 Epithelial remodeling and epithelial-mesenchymal transition

Repeated or prolonged epithelial injury, together with impaired repair processes, leads to structural alterations in the airway that are collectively defined as airway remodeling. These changes include thickening of the reticular basement membrane, goblet cell hyperplasia, subepithelial fibrosis, and airway smooth muscle cell (ASMC) hyperplasia and hypertrophy (30, 83, 84) (**Figure 2**). Epithelial cells derived from patients with asthma exhibit delayed and dysregulated repair responses following injury. This impaired regeneration is characterized by asynchronous cell proliferation and an inability to rapidly restore a continuous and functionally competent epithelial layer (85).

Under conditions of persistent inflammation, epithelial cells may undergo epithelial–mesenchymal transition (EMT), a process in which epithelial characteristics such as apicobasal polarity and intercellular junctions are progressively lost, accompanied by acquisition of mesenchymal features including increased migratory capacity and altered secretory profiles (86). This process is largely driven by transforming growth factor-β (TGF-β), which is produced by both injured epithelial cells and infiltrating immune cells. In diseases such as silicosis and severe steroid-resistant asthma, single-cell transcriptomic and pseudotime analyses have identified stress-associated epithelial subpopulations exhibiting EMT-like features that contribute to immune activation and tissue remodeling (87). Through EMT, epithelial cells contribute directly to the expansion of subepithelial myofibroblasts and promote the deposition of extracellular matrix (ECM) components. This process results in progressive narrowing of the airway lumen and increased stiffness of lung tissue, ultimately leading to irreversible structural changes (84, 88) (**Figure 2**).

## 4 Molecular mechanisms of epithelium-driven inflammation

### 4.1 Release and function of alarmins

**Figure 3:**
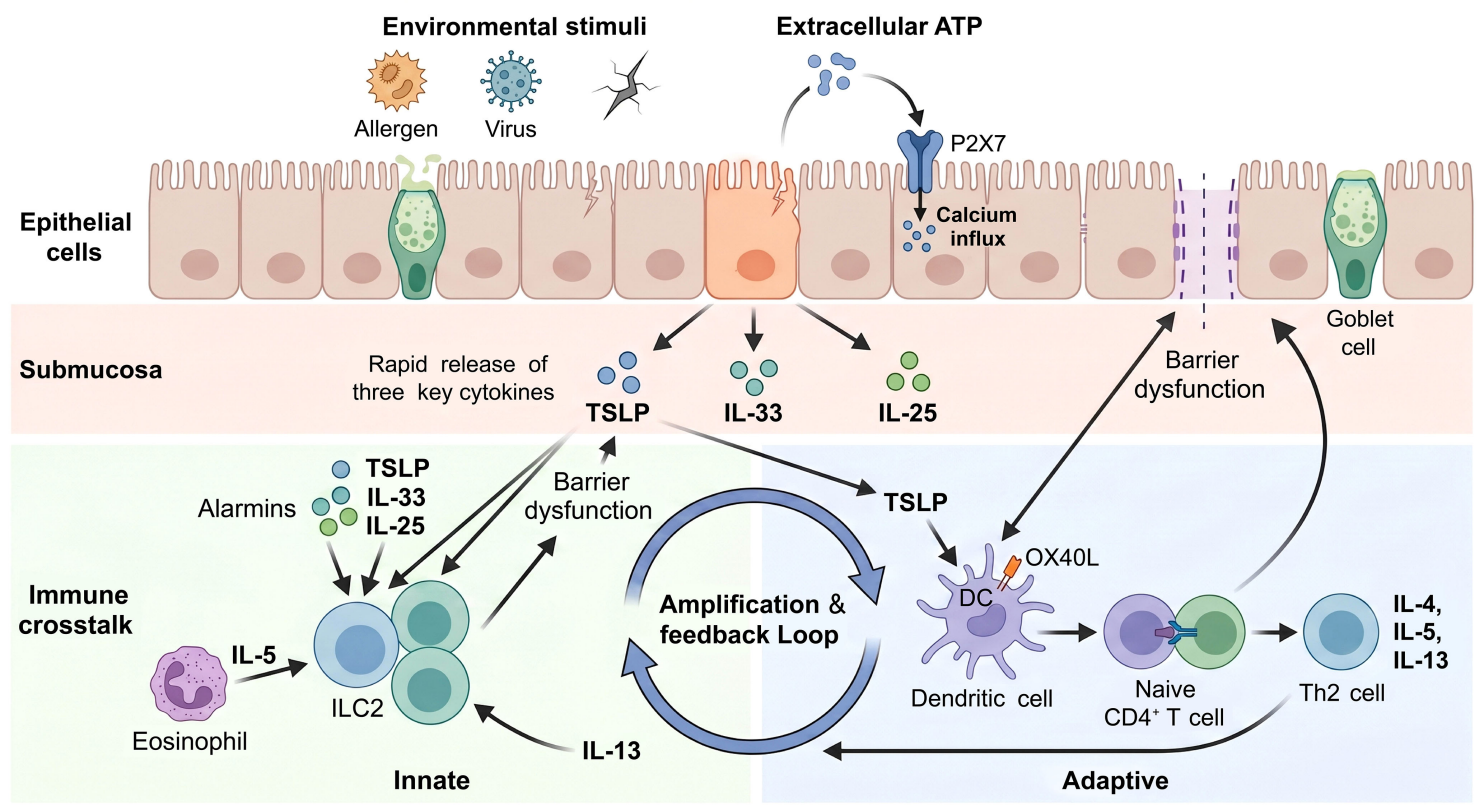
Mechanisms of epithelium-driven inflammation and immune crosstalk. Upon exposure to environmental stimuli such as allergens or respiratory viruses, injured airway epithelial cells release extracellular ATP. This ATP activates purinergic receptors (e.g., P2X7), leading to calcium influx and the rapid release of key epithelial alarmins (TSLP, IL-33, and IL-25) into the submucosa. These alarmins initiate parallel immune cascades. In the innate immune pathway (left), alarmins activate type 2 innate lymphoid cells (ILC2s) to secrete IL-5, which recruits eosinophils, and IL-13. In the adaptive immune pathway (right), TSLP conditions dendritic cells by upregulating OX40L, promoting the differentiation of naive CD4⁺ T cells into Th2 cells that produce IL-4, IL-5, and IL-13. Ultimately, the accumulation of these type 2 cytokines drives an amplification and feedback loop (center), further exacerbating epithelial barrier dysfunction, promoting goblet cell hyperplasia, and sustaining chronic airwayinflammation.

At the epithelial–immune interface, epithelial-derived alarmins play a central role in initiating inflammatory responses. These include thymic stromal lymphopoietin (TSLP), interleukin-33 (IL-33), and interleukin-25 (IL-25), which are recognized as key upstream mediators in the early phase of airway inflammation (3, 89, 90). These cytokines are rapidly released in response to epithelial stress signals, including mechanical injury, exposure to protease-containing allergens, and viral infection. Their secretion is tightly controlled by intracellular sensing mechanisms. Allergen exposure, for example, induces the release of extracellular ATP, which functions as a damage-associated molecular signal. This ATP activates purinergic receptors such as P2X_7_, leading to increased intracellular calcium levels and subsequent secretion of IL-33, TSLP, and IL-25 (3, 91–93) (**Figure 3**). Following release, these alarmins exert broad effects on multiple cell types. IL-33 signals through its receptor ST2 (IL1RL1), which is expressed on various immune cells, including eosinophils, neutrophils, macrophages, and mast cells. Activation of this pathway leads to downstream signaling through NF-κB and mitogen-activated protein kinase (MAPK) pathways (94, 95). IL-25, which is stored intracellularly in epithelial cells and can be rapidly released upon stimulation, signals through a heterodimeric receptor complex involving IL-17RB and IL-17RA, with IL-17RB serving as the IL-25-associated receptor subunit and IL-17RA contributing to functional signaling (96–98). TSLP exhibits a broader range of target interactions. In addition to acting on immune cells, it also influences sensory neurons and airway smooth muscle cells, thereby linking epithelial signaling with neuroimmune and structural cell responses. Through these interactions, TSLP contributes to bronchoconstriction and airway hyperresponsiveness (99, 100).

### 4.2 Epithelial-innate immune cell crosstalk

Alarmins released by epithelial cells function as key regulatory signals that shape innate immune responses. Among the primary responders are group 2 innate lymphoid cells (ILC2s), which are highly sensitive to epithelial-derived cytokines. IL-25 and other epithelial alarmins directly stimulate ILC2s, promoting expansion and production of type 2 cytokines (101). In airway type 2 inflammation, activated pulmonary ILC2s are emphasized mainly as sources of IL-5 and IL-13, whereas IL-9 functions as an important autocrine survival and amplification factor and IL-4 may be produced under selected conditions (102–104). IL-5 plays a major role in promoting eosinophil recruitment, survival, and activation, leading to local eosinophilic inflammation (**Figure 3**). At the same time, IL-13 acts on epithelial cells to induce goblet cell differentiation and mucus production, thereby altering airway structure and function. This IL-9-dependent survival signal further contributes to this process and can indirectly promote mast cell activation through induction of IL-2 release (3, 104–106). Together, these interactions define a coordinated innate immune network regulated by epithelial signals. In parallel, epithelial cells regulate the activity of dendritic cells (DCs), which serve as essential intermediaries between innate and adaptive immunity. Upon sensing environmental stimuli, epithelial cells produce cytokines and chemokines that recruit and activate DCs (58). Among these, TSLP plays a particularly important role by conditioning airway-resident DCs to upregulate co-stimulatory molecules, including OX40 ligand (OX40L) (107) (**Figure 3****).** The interaction between OX40L-expressing DCs and OX40 on naïve CD4⁺ T cells within draining lymph nodes represents a critical step in promoting Th2 differentiation. This process is associated with suppression of regulatory T cell function and the establishment of long-term allergic immune responses (107).

### 4.3 Epithelial-adaptive immune cell crosstalk

Signals originating from the airway epithelium extend into the adaptive immune system through coordinated interactions involving dendritic cells, T cells, and B cells (108). Following antigen uptake and processing, TSLP-conditioned dendritic cells migrate to lymphoid tissues, where they present antigens to naïve CD4⁺ T cells and promote their differentiation into Th2 cells. Activated Th2 cells subsequently migrate to the airway mucosa and produce cytokines including IL-4, IL-5, and IL-13. These cytokines collectively drive key features of allergic inflammation, including eosinophil recruitment, immunoglobulin class switching, B cell activation, goblet cell differentiation, and mucus production (3, 109–111). Interactions between epithelial-derived cytokines, Th2 cells, and B cells are essential for the development of allergic sensitization. In particular, IL-4, together with epithelial-derived signals, promotes class switch recombination in B cells, resulting in the production of allergen-specific IgE. IgE binds to the high-affinity FcϵRI receptor on mast cells and basophils. Upon subsequent allergen exposure, cross-linking of receptor-bound IgE induces rapid degranulation and release of mediators such as histamine. This process contributes to further epithelial injury and reinforces ongoing inflammatory responses (82, 112).

### 4.4 Inflammatory amplification and positive feedback regulatory networks

A defining feature of the airway epithelial–immune axis is the presence of self-reinforcing regulatory circuits that sustain and amplify inflammatory responses. These feedback mechanisms play a critical role in the transition from acute responses to chronic disease states (113). One representative example involves the interaction between epithelial-derived TSLP and type 2 cytokines. Initial TSLP release activates ILC2s and Th2 cells, leading to increased production of IL-13. IL-13 then acts on epithelial cells through the IL-4Rα/IL-13Rα1 receptor complex, resulting in suppression of barrier-associated proteins such as claudin-18, increased production of periostin, and enhanced expression of eosinophil-attracting chemokines such as CCL11 (21, 114) (**Figure 3**). In addition, IL-13 further promotes TSLP production, thereby reinforcing the inflammatory loop. Recent studies have also shown that TSLP can promote the formation of extracellular traps by eosinophils and regulate basophil activation, which in turn enhances TSLP production and further amplifies airway inflammation (115). Through these interconnected pathways, epithelial barrier integrity is progressively compromised, allowing increased penetration of environmental stimuli. This results in sustained activation of epithelial cells and continuous release of inflammatory mediators. Such self-perpetuating cycles help explain the persistence and treatment resistance observed in chronic airway diseases and underscore the importance of targeting upstream regulatory mechanisms within the epithelial–immune axis.

## 5 Role of epithelium-driven inflammation in respiratory diseases

### 5.1 Asthma

Asthma exemplifies how epithelial dysfunction can drive airway inflammation (18). It is characterized by a predominant type 2 immune response, and increasing evidence indicates that excessive production of epithelial-derived alarmins contributes to disease exacerbations. Current understanding supports the concept that asthma originates, at least in part, from intrinsic defects in epithelial barrier function. Impaired barrier integrity results in increased susceptibility to environmental exposures and facilitates sustained immune activation (20). This perspective has contributed to the emergence of an epithelium-centered framework in asthma research, in which epithelial abnormalities are considered fundamental across different disease endotypes. T2-high asthma is typically associated with eosinophilic inflammation and corticosteroid responsiveness, driven by canonical alarmin release and epithelial dysfunction (19). In contrast, a subset of severe (often misclassified as T2-low) asthma is characterized by neutrophilic inflammation and corticosteroid resistance, primarily driven by T1/IFN-γ signaling and tissue-resident memory T cells, featuring epithelial disruption but distinct from the classic T2-alarmin axis (116). Structural and functional alterations in the airway epithelium include impaired repair capacity, persistent disruption of tight junctions, and increased baseline production of TSLP, IL-33, and IL-25. These epithelial-derived signals contribute to key pathological features of asthma, including airway hyperresponsiveness (AHR), mucus overproduction, and subepithelial fibrosis (117). The NLRP3-dependent barrier homeostasis described above further supports this view, because impaired NLRP3 signaling can reduce junctional molecule expression and enhance allergen uptake and AHR in experimental allergic airway inflammation (22).

### 5.2 Chronic obstructive pulmonary disease

Chronic obstructive pulmonary disease (COPD) has traditionally been viewed as a macrophage- and neutrophil-driven disorder linked to protease–antiprotease imbalance. However, recent evidence indicates that epithelial dysfunction is a major contributor to disease pathogenesis. Chronic exposure to cigarette smoke and environmental pollutants induces persistent epithelial injury, leading to altered differentiation, cellular senescence, and impaired regenerative capacity (23, 118). Organoid-based studies combined with single-cell RNA sequencing have demonstrated that bronchial epithelial cells from patients with COPD exhibit disrupted differentiation trajectories, expansion of basal/suprabasal-like cell populations, and impaired epithelial homeostasis, accompanied by goblet cell hyperplasia and reduced ciliated-cell differentiation (24). The pattern of epithelial-derived signaling in COPD differs from that observed in allergic asthma. In particular, cigarette smoke exposure is associated with altered IL-33 signaling, which is linked to a shift away from type 2 inflammation toward type 1–dominant immune response (25). IL-33 contributes to the activation of macrophages and neutrophils through ST2-dependent pathways, thereby promoting neutrophilic inflammation and tissue damage. In addition, sustained epithelial stress leads to continuous production of transforming growth factor-β (TGF-β), which contributes to small airway fibrosis and progressive decline in lung function, including reductions in forced expiratory volume (FEV1) (26, 119).

### 5.3 Upper airway inflammatory diseases

The unified airway hypothesis proposes that the upper and lower respiratory tracts constitute a continuous immunological and functional unit characterized by shared epithelial, inflammatory, and immune mechanisms (28). Within this framework, epithelial dysfunction in the nasal and sinus mucosa is closely linked to pathological processes in the lower airways. In allergic rhinitis and chronic rhinosinusitis (CRS), particularly CRS with nasal polyps (CRSwNP), epithelial barrier disruption and increased alarmin production closely resemble the alterations observed in asthma (29). TSLP plays a central role in mediating interactions between epithelial cells and immune cells and contributes to the amplification of type 2 inflammatory responses in CRS (30, 120, 121). Sustained release of epithelial-derived cytokines promotes local eosinophilic inflammation and tissue remodeling, including the formation and growth of nasal polyps. Beyond their local effects in the airway mucosa, epithelial-derived alarmins can exert systemic immunomodulatory functions and contribute to airway–bone marrow crosstalk, including the mobilization of hematopoietic progenitor cells (122). Epithelial barrier dysfunction in the upper airway has also been implicated as a contributing factor in the progression of inflammation from the upper to the lower respiratory tract (31, 123, 124). This provides a mechanistic basis for the clinical association between allergic rhinitis and the development or exacerbation of asthma.

### 5.4 Infection-related respiratory diseases

Respiratory viral infections, particularly during early life, can significantly influence the development of the epithelial–immune axis and increase susceptibility to chronic respiratory diseases (125). Respiratory syncytial virus (RSV), a major cause of bronchiolitis in infants, disrupts epithelial structure and function and induces the release of alarmins, including IL-33. Elevated levels of IL-33 have been detected in nasal secretions of children with RSV infection and are associated with the induction of type 2 inflammatory responses (37, 126, 127). Viral infections also promote the release of damage-associated molecules, such as HMGB1, through mechanisms involving reactive oxygen species. In addition, accumulating evidence suggests that viral exposure can induce long-lasting epigenetic alterations in epithelial progenitor cells, particularly basal cells. These changes may modify epithelial differentiation and immune responsiveness, resulting in a lower threshold for type 2 inflammation upon subsequent exposure to environmental allergens (36, 128). Similar mechanisms have been observed in infections caused by human metapneumovirus (hMPV), where elevated TSLP levels have been associated with recurrent wheezing and poor asthma control in affected children (38).

## 6 Therapeutic strategies targeting the epithelial-immune axis

### 6.1 Biologics targeting alarmins

**Figure 4:**
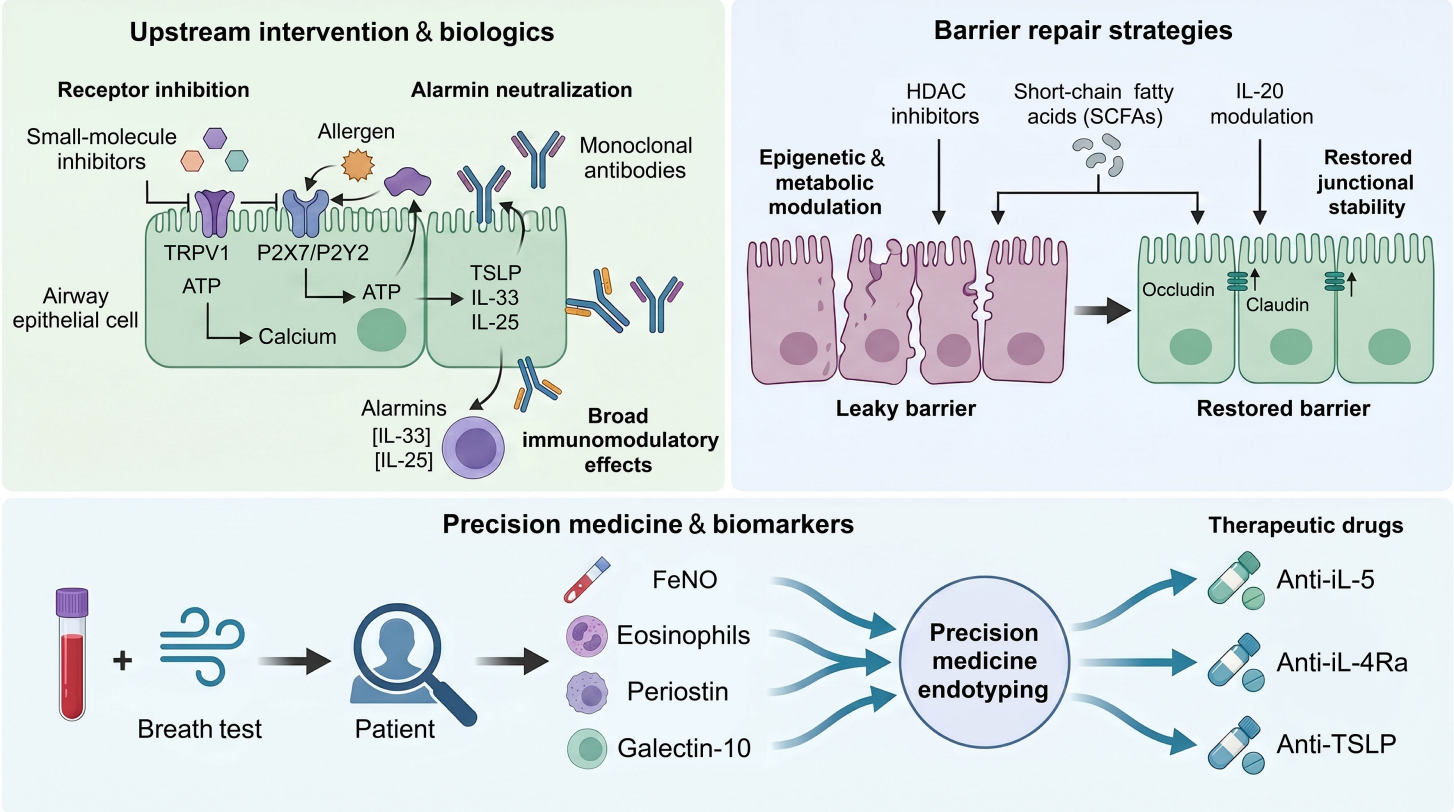
Therapeutic strategies and precision medicine targeting the airway epithelial-immune axis. This schematic illustrates three main clinical approaches: (top left) upstream interventions: small-molecule inhibitors block epithelial sensory receptors (e.g., TRPV1, P2X7/P2Y2), while monoclonal antibodies neutralize key epithelial alarmins (TSLP, IL-33, IL-25) to attenuate downstream inflammation. (top right) barrier repair strategies: epigenetic, post-translational (e.g., CBX4-mediated SUMOylation), metabolic (e.g., HDAC inhibitors, SCFAs), and cytokine (IL-20) modulators restore junctional stability in leaky barriers. (Bottom) precision medicine: clinical biomarkers (FeNO, eosinophils, periostin, Galectin-10) guide precise endotyping to optimize the selection of targeted biologics (anti-IL-5, anti-IL-4Rα, anti-TSLP).

As upstream initiators of epithelial-driven inflammation, alarmins such as TSLP, IL-33, and IL-25 have attracted considerable interest as therapeutic targets, leading to the development of biologic agents aimed at broadly modulating chronic airway inflammation (**Figure 4**) (129, 130). These cytokines function at an early stage of the inflammatory cascade, and their inhibition provides an opportunity to modulate upstream immune activation (131, 132). Targeting alarmins offers potential advantages over therapies directed at downstream type 2 cytokines such as IL-4, IL-5, and IL-13. By intervening at an earlier point in the signaling hierarchy, these biologics may exert broader immunomodulatory effects and influence multiple inflammatory pathways simultaneously (133). This approach is particularly relevant for patients with non–T2-high disease phenotypes, including neutrophilic and paucigranulocytic asthma, which often show limited responsiveness to inhaled corticosteroids and existing biologic therapies. Overall, inhibition of alarmin signaling represents an important therapeutic direction, with the potential to address unmet clinical needs across heterogeneous disease endotypes. However, alarmin-directed therapies differ substantially in clinical maturity. Anti-TSLP therapy with tezepelumab is already approved as add-on maintenance treatment for severe asthma and is supported by phase 3 clinical evidence, whereas IL-33/ST2-directed approaches such as astegolimab remain clinical trial candidates, and IL-25-directed strategies are still mainly preclinical or early translational (131, 134, 135).

### 6.2 Epithelial barrier repair strategies

In addition to controlling inflammation, restoration of epithelial barrier integrity represents a key objective for achieving sustained disease modification. Increasing attention has been directed toward strategies that enhance junctional stability and correct underlying regulatory abnormalities affecting epithelial function. In this context, NLRP3-dependent epithelial barrier homeostasis may represent a mechanistically relevant node, because loss of NLRP3 signaling can weaken junctional molecule expression and promote allergen penetration in experimental allergic airway inflammation (22). Epigenetic mechanisms, including DNA methylation, histone modifications, and non-coding RNA–mediated regulation, are increasingly recognized as important determinants of epithelial barrier integrity through modulation of barrier-associated genes such as FLG (136, 137). Environmental exposures, including particulate matter such as PM2.5, can induce aberrant epigenetic modifications that contribute to junctional disruption (138). In addition, SUMOylation represents an important post-translational modification relevant to epithelial barrier repair. Liang et al. reported that blockade of CBX4-mediated beta-catenin SUMOylation attenuated airway epithelial barrier dysfunction in asthma, suggesting that dysregulated SUMOylation may connect epithelial stress signaling with junctional instability (139). Pharmacological modulation of epigenetic pathways has therefore been explored as a potential therapeutic approach. Histone deacetylase (HDAC) inhibitors have been shown to improve epithelial barrier function in preclinical models (**Figure 4**). For example, treatment of primary human nasal epithelial cells (HNECs) with the HDAC inhibitor JNJ-26481585 increases occludin expression and enhances transepithelial electrical resistance (TEER), indicating improved barrier integrity (140, 141). Additional strategies focus on modulation of the local metabolic environment. Short-chain fatty acids, including propionate and butyrate, have been reported to restore allergen-induced barrier dysfunction in bronchial epithelial cells, suggesting potential therapeutic relevance in asthma (31). Targeting cytokines involved in epithelial repair processes also represents a promising direction. Modulation of the IL-20 cytokine family has been shown to improve epithelial regeneration in experimental models of COPD, indicating that selective immunomodulation may support restoration of epithelial structure and function (142).

### 6.3 Inflammatory signaling pathway interventions

In addition to biologic therapies, small-molecule inhibitors targeting epithelial sensing and signaling pathways are under active investigation. These approaches aim to interfere with early signaling events that precede the activation of downstream immune responses. Purinergic signaling has been identified as an important regulator of epithelial activation. Allergen-induced release of extracellular ATP activates purinergic receptors such as P2X_7_ and P2Y_2_, leading to intracellular calcium signaling that is required for the release of alarmins, including IL-33 and TSLP (93; 143). Inhibition of these receptors has been shown to attenuate epithelial activation and reduce downstream inflammatory responses (**Figure 4**). Transient receptor potential (TRP) channels, particularly TRPV1, have been implicated in epithelial sensing of environmental stimuli. Pharmacological inhibition or genetic silencing of TRPV1 has been shown to attenuate protease allergen-induced ATP release and IL-33 secretion in airway epithelial cells (144). Targeting these upstream signaling pathways may provide an opportunity to limit the initiation of inflammatory responses at an early stage, before extensive immune activation occurs.

### 6.4 Precision medicine and biomarkers

The clinical heterogeneity of asthma and COPD underscores the need for precision medicine approaches guided by reliable biomarkers. Classification of disease endotypes based on underlying mechanisms is essential for optimizing therapeutic selection. Currently, peripheral blood eosinophil counts, sputum eosinophils, and fractional exhaled nitric oxide (FeNO) are widely used as accessible indicators of type 2 inflammation and are routinely applied in guiding biologic therapies targeting IL-5 and IL-4Rα pathways (**Figure 4**) (145, 146). Emerging biomarkers provide additional insight into epithelial activation and airway remodeling. Serum periostin has been shown to correlate with multiple indicators of type 2 inflammation, including eosinophil levels and immunoglobulin E (IgE), reflecting IL-13–driven epithelial responses (147–149). Galectin-10, a major component of Charcot–Leyden crystals, has been identified as a marker of eosinophil extracellular trap formation and is associated with small airway dysfunction. Changes in its levels may also reflect responses to targeted therapies (146, 150). Integration of multiple biomarker profiles is expected to improve patient stratification and enable selection of therapies targeting specific components of the epithelial–immune axis, thereby enhancing treatment efficacy.

## 7 Emerging technologies and research frontiers

### 7.1 Single-cell RNA sequencing

**Figure 5:**
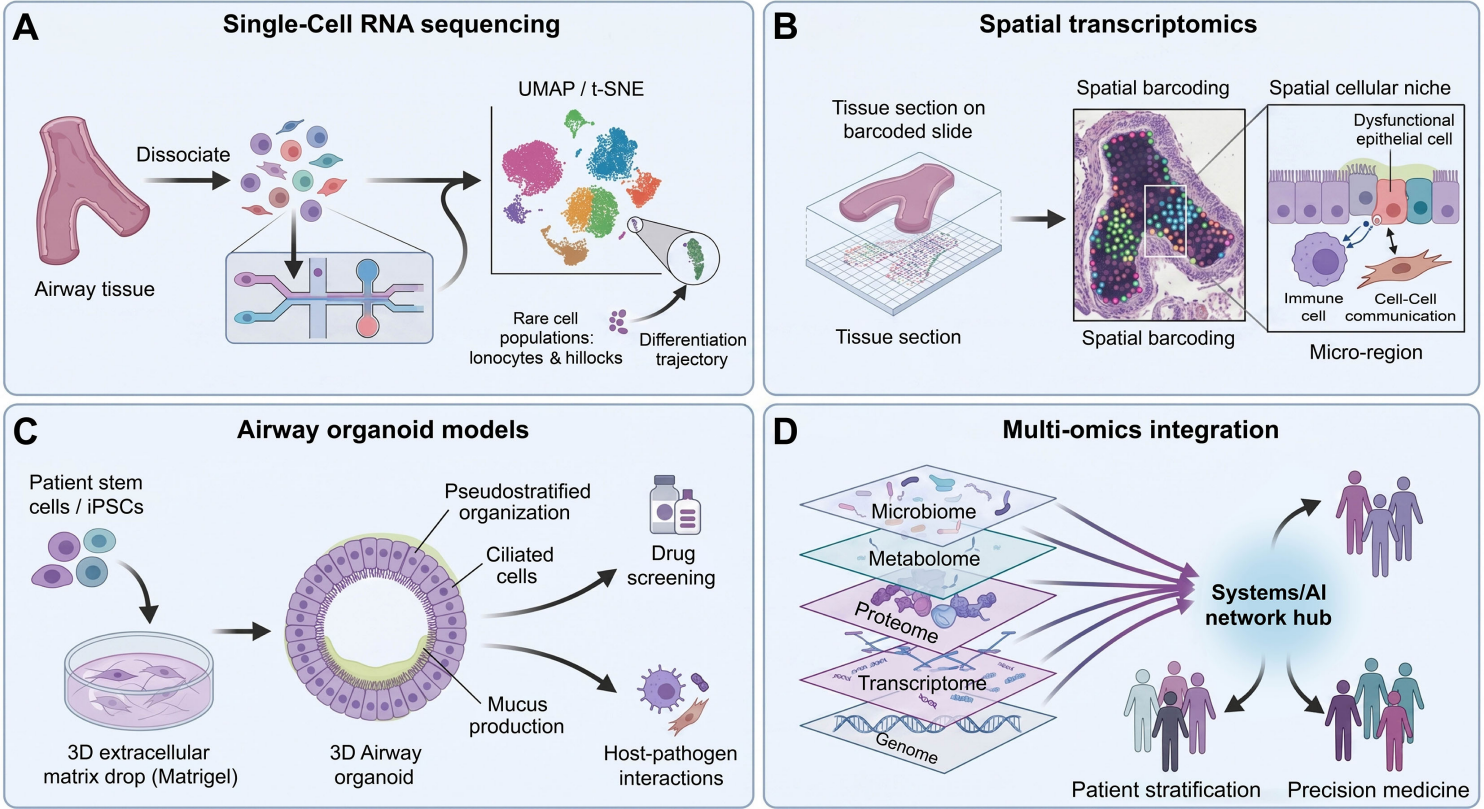
Emerging technologies for investigating the airway epithelial-immune axis. **(A)** Single-cell RNA sequencing (scRNA-seq): Dissociation of airway tissue enables high-resolution transcriptomic profiling, facilitating the identification of rare cell populations (e.g., ionocytes, hillocks) and differentiation trajectories. **(B)** Spatial transcriptomics: preserves spatial gene expression contexts within tissue sections, allowing for the mapping of cellular niches and cell-cell communication networks in defined micro-regions. **(C)** Airway organoid models: patient-derived stem cells or iPSCs cultured in 3D matrices self-organize into pseudostratified epithelia, providing robust *in vitro* platforms for drug screening and modeling host-pathogen interactions. **(D)** Multi-omics integration: combines multi-layered biological datasets (genome, transcriptome, proteome, metabolome, and microbiome) via systems biology approaches to advance patient stratification and precision medicine.

The application of single-cell RNA sequencing (scRNA-seq) has substantially advanced the understanding of cellular heterogeneity within the respiratory tract. By enabling transcriptomic profiling at single-cell resolution, this approach overcomes the limitations of bulk tissue analysis and allows the identification of rare but functionally important cell populations (151, 152) (**Figure 5A**). Using scRNA-seq, previously unrecognized epithelial cell types have been identified, including FoxI1-positive pulmonary ionocytes and distinct epithelial populations located in high-turnover squamous structures termed “hillocks” (45). These findings have important implications for airway biology. For example, ionocytes have been shown to represent a major site of CFTR expression, providing new insights into cystic fibrosis pathogenesis, while hillock-associated cells may contribute to epithelial maintenance and regeneration following injury (50, 153). In addition, scRNA-seq has enabled reconstruction of epithelial differentiation trajectories, revealing transitional cellular states that are altered in disease. In COPD, expansion of suprabasal epithelial cell populations has been observed and is associated with impaired differentiation and defective repair processes (154). By linking cell type–specific transcriptional programs with disease-associated genes, scRNA-seq provides a framework for understanding how epithelial dysfunction contributes to respiratory disease at a cellular level.

### 7.2 Spatial transcriptomics

While scRNA-seq identifies cellular composition, spatial transcriptomics preserves the spatial organization of gene expression within tissue, thereby providing critical contextual information regarding cell–cell interactions (**Figure 5B**). Recent studies applying spatial transcriptomics to airway tissues have demonstrated that inflammatory processes are not uniformly distributed but instead occur within defined microenvironments (155–157). These analyses have identified localized cellular niches characterized by coordinated expression of chemokines, alarmins, and signaling molecules. Within these spatially restricted regions, epithelial cells interact with immune and stromal cells to form functional units that regulate inflammatory responses. For example, in conditions such as severe asthma and fibrotic lung disease, these niches may be enriched with epithelial cells exhibiting stress-associated or epithelial–mesenchymal transition (EMT)-like features, alongside specific fibroblast and immune cell subsets (87, 157, 158). Mapping these spatial relationships provides insight into how localized epithelial dysfunction drives tissue-level pathology and may support the development of therapies targeting disease-relevant microenvironments.

### 7.3 Organoid models

Conventional two-dimensional cell cultures and animal models have important limitations in recapitulating human airway biology. Organoid systems have emerged as advanced *in vitro* platforms that more closely mimic the structural and functional properties of native tissues (159). Airway organoids can be generated from adult stem cells, embryonic stem cells, or induced pluripotent stem cells (iPSCs), and they exhibit key features of the airway epithelium, including pseudostratified organization, ciliary function, and mucus production (160–162) (**Figure 5C**). These systems retain the capacity for self-renewal and self-organization, allowing the study of epithelial dynamics under controlled conditions. Organoid models have been widely applied to investigate disease mechanisms, including genetic disorders such as cystic fibrosis, as well as host–pathogen interactions. For instance, they have been used to study viral tropism and epithelial responses during infections, including those caused by respiratory viruses (163–165). In addition, organoids provide a platform for drug screening and personalized medicine approaches, enabling the evaluation of therapeutic responses in patient-derived tissues prior to clinical application (166).

### 7.4 Multi-omics integration

Advances in high-throughput technologies have enabled the integration of multiple layers of biological data, supporting a systems-level understanding of respiratory disease. Multi-omics approaches combine genomic, transcriptomic, proteomic, metabolomic, and microbiome data to characterize interactions between the airway epithelium, immune system, and microbial environment (167) (**Figure 5D**). Integration of these datasets allows identification of complex regulatory networks and improves the ability to stratify patients based on underlying biological mechanisms. Studies incorporating multi-omic analyses have demonstrated that distinct patient subgroups can be defined by combined molecular signatures, including microbial composition, inflammatory mediators, and epithelial gene expression profiles (27, 168–170). These approaches also facilitate the identification of signaling pathways involved in host–pathogen interactions, including kinase pathways activated in response to bacterial toxins. By reducing the limitations associated with individual data types, multi-omics integration supports the discovery of composite biomarkers and potential therapeutic targets, contributing to the advancement of precision medicine in respiratory diseases (171).

## 8 Conclusion and perspectives

The conceptual framework of respiratory disease has undergone a significant shift, with the airway epithelium now recognized as an active regulator of mucosal immunity rather than a passive physical barrier. Increasing evidence supports the view that the epithelial–immune axis represents a central interface through which environmental exposures, including allergens, pathogens, and pollutants, are translated into sustained inflammatory responses. Through the release of epithelial-derived mediators such as TSLP, IL-33, and IL-25, and the subsequent activation of both innate and adaptive immune pathways, epithelial dysfunction contributes to the initiation and maintenance of chronic airway diseases, including asthma, COPD, and chronic rhinosinusitis. Within this framework, epithelial alterations are not merely secondary consequences of inflammation but can act as key drivers within reciprocal epithelial-immune feedback loops. This evolving understanding has important therapeutic implications. Targeting upstream mediators, particularly alarmins, has emerged as a promising strategy for modulating multiple inflammatory pathways simultaneously, with potential applicability across heterogeneous disease endotypes. In parallel, approaches aimed at restoring epithelial barrier integrity, including modulation of epigenetic regulators and metabolic pathways, offer opportunities for achieving more sustained disease control beyond symptom suppression.

Despite this progress, several challenges still limit the clinical translation of epithelial-targeted therapies. First, the pronounced heterogeneity of airway diseases complicates patient stratification and the selection of optimal therapeutic strategies (172). Second, current biomarkers, although clinically informative, do not fully reflect the complexity of epithelial dysfunction or its dynamic interactions with the immune system and microbiome (173). Third, the long-term efficacy and safety of therapies targeting upstream pathways, including alarmins and epithelial repair mechanisms, remain to be validated across diverse patient populations (174). Addressing these challenges requires targeted future research. Integrating multi-layered datasets from single-cell transcriptomics, spatial profiling, and organoid-based functional studies will provide a comprehensive map of epithelial–immune interactions across disease states. Developing robust, non-invasive biomarkers capable of accurately reflecting epithelial status is essential for enabling precision medicine approaches. Finally, elucidating the mechanisms governing epithelial repair and their dysregulation will inform the design of therapies aimed at restoring tissue homeostasis rather than solely suppressing inflammation. Collectively, these efforts are expected to improve patient stratification, guide targeted interventions, and advance long-term disease-modifying strategies (175–177). Ultimately, deeper understanding of the airway epithelial–immune axis may reshape disease classification and guide more targeted, individualized therapies. Continued integration of mechanistic insights with translational and clinical research will be essential for realizing this goal.
